# Identification of a competing endogenous RNA axis “SVIL‐AS1/miR‐103a/ICE1” associated with chemoresistance in lung adenocarcinoma by comprehensive bioinformatics analysis

**DOI:** 10.1002/cam4.4132

**Published:** 2021-07-15

**Authors:** Lili Guo, Lina Ding, Junfang Tang

**Affiliations:** ^1^ Department of Medical Oncology Beijing Tuberculosis and Thoracic Tumor Research Institute Beijing Chest Hospital Capital Medical University Beijing China; ^2^ Key Laboratory of Henan Province for Drug Quality and Evaluation School of Pharmaceutical Sciences Ministry of Education of China Zhengzhou University Zhengzhou P.R. China

**Keywords:** chemoresistance, competing endogenous RNA, lung adenocarcinoma, prognosis, weighted gene co‐expression network analysis

## Abstract

**Background:**

Chemotherapy is an important treatment for lung cancer. The molecular mechanism of lung adenocarcinoma (LUAD) chemoresistance is not completely understood.

**Methods:**

Weighted gene co‐expression network analysis (WGCNA) was applied to screen the modules related to chemosensitivity using the data of LUAD patients receiving chemotherapy in The Cancer Genome Atlas database. GDCRNATools package was used to establish competing endogenous RNA (ceRNA) network based on the key chemotherapy‐related module. Kaplan–Meier and risk models were used to analyze the influence of genes in the ceRNA network on the prognosis of LUAD patients receiving chemotherapy. Cell counting kit‐8, reverse transcription‐quantitative PCR, and dual‐luciferase reporter assay were used to detect the effects of abnormal expression of genes in the ceRNA network on the proliferation and IC50 of cisplatin (DDP)‐resistant LUAD cells, and the targeting relationship of genes in the ceRNA network. The signaling pathways and functions of ICE1 in LUAD were analyzed by LinkOmics and CancerSEA databases, and validated by Western blot.

**Results:**

Midnightblue module was the only WGCNA module positively correlated with chemosensitivity, in which the function of genes was related to cancer progression. SVIL‐AS1/miR‐103a/ICE1 was constructed based on midnightblue module. High expression of SVIl‐AS1 and ICE1 corresponded to a favorable prognosis. High expression of miR‐103a corresponded to a dismal prognosis. SVIl‐AS1 was downregulated in DDP‐resistant LUAD cells. SVIL‐AS1 overexpression retarded the proliferation and DDP resistance of DDP‐resistant LUAD cell. miR‐103a was sponged by SVIL‐AS1 and directly targeted ICE1. miR‐103a overexpression and ICE1 knockdown overturned the suppressive effect of SVIL‐AS1 overexpression on cell proliferation and DDP resistance. Further bioinformatics analysis and experimental verification showed that SVIL‐AS1/miR‐103a‐3p/ICE1 axis can enhance DNA damage caused by chemotherapeutic agents.

**Conclusions:**

SVIL‐AS1 inhibited chemoresistance by acting as a sponge for miR‐103a and upregulating ICE1 expression, which may be a potential therapeutic target for chemotherapy in LUAD.

## Graphical abstract

The present study identified a novel competing endogenous RNA axis (SVIL‐AS1/miR‐103a/ICE1) that regulated lung adenocarcinoma (LUAD) chemotherapy resistance. SVIL‐AS1, miR‐103a, and ICE1 can be used as independent risk factors for the prognosis of LUAD patients with chemotherapy.

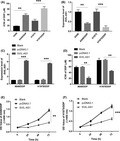

## 1 INTRODUCTION

Lung cancer is a malignant tumor with the highest morbidity and mortality in the world, among which non‐small cell lung cancer (NSCLC) accounts for about 85%.^1^, ^2^ As a common pathological type of NSCLC, lung adenocarcinoma (LUAD) has strong infiltration and metastasis.^3^ The early symptoms of LUAD are atypical. Most of LUAD patients are diagnosed with middle and advanced stage. The 5‐year survival rate of LUAD is only about 15%.^4^ Chemotherapy is one of the main treatment options for lung cancer.^5^, ^6^ However, lung cancer patients receiving chemotherapy are prone to chemoresistance after long‐term medication, leading to treatment failure. Cisplatin (DDP)‐based chemotherapy is the mainstream chemotherapy for lung cancer patients.^7^, ^8^ Once patients are resistant to DDP‐based chemotherapy, there will be very few other chemotherapy options.^7^, ^8^ The mechanism of chemoresistance in lung cancer is still poorly understood. Therefore, it is urgent to explore the chemoresistance mechanism to improve the diagnosis and treatment of LUAD.

Chemoresistance of LUAD is a complex biological process regulated by multiple genes. As a systematic biology method based on scale‐free network distribution, weighted gene co‐expression network analysis (WGCNA) can visually show the relationship among various parts of a biological system.^9^, ^10^ Compared with previous studies of individual genes, WGCNA displays the characteristics of biological systems more precisely. By dividing genes into different modules for functional prediction, WGCNA can explore the potential relationship between gene networks and clinical traits.^9^, ^10^ As a powerful bioinformatics tool for the identification of cancer‐related genes, WGCNA is widely used in the research of gastric cancer, breast cancer, bladder cancer, and other malignant tumors.^11^, ^12^, ^13^ The application of WGCNA to explore LUAD chemoresistance will provide new ideas for advancing the research of LUAD regulation mechanism.

Long non‐coding RNAs (lncRNAs) is a class of non‐coding RNA with a length of >200 nucleotides.^14^, ^15^ lncRNAs play a regulatory role at various levels, such as chromatin modification, transcriptional regulation, post‐protein translation regulation, and microRNA (miRNA) regulation.^16^, ^17^, ^18^ Competing endogenous RNA (ceRNA) hypothesis is an important mechanism by which lncRNAs regulate gene expression.^19^ lncRNA competitively blocks miRNA to relieve the inhibitory effect of miRNA on target genes, thereby promoting the expression of target genes.^19^ lncRNA MT1JP acts as a ceRNA to regulate FBXW7 by competitively binding miR‐92a‐3p to inhibit gastric cancer progression.^20^ lncRNA ZEB2‐AS1 sponges miR‐204 to promote pancreatic cancer progression.^21^ lncRNA LCAT1 acts as a ceRNA to regulate miR‐4715‐5p/RAC1/PAK1 axis and promote the development of lung cancer.^22^ However, lncRNA regulates LUAD chemoresistance through ceRNA mechanism has not been clearly studied.

In the present study, WGCNA was applied to screen modules related to chemosensitivity in LUAD patients. Function of genes in the key chemotherapy‐related module was annotated. A ceRNA axis based on the key chemotherapy‐related module was constructed. The influence of genes in the ceRNA axis on the prognosis of LUAD patients was analyzed. Finally, the targeting relationship and biological function of genes in ceRNA axis were verified at the cellular level. The results of this study not only contribute to the theoretical study of ceRNA regulation of chemoresistance in LUAD, but also provide potential biomarkers for LUAD chemotherapy.

## 2 METHODS

### 2.1 Data collection

RNA expression profiles and clinical data of LUAD patients receiving chemotherapy were collected from The Cancer Genome Atlas (TCGA) and Kaplan–Meier plotter databases. The dataset of TCGA (including 133 LUAD patients receiving chemotherapy) was used for WGCNA, overall survival analysis, risk model analysis, and Pearson's correlation analysis of SVIL‐AS1, miR‐103a‐3p, and ICE1. Single‐cell sequencing data of LUAD cells was downloaded from Cancer Single‐cell State Atlas (CancerSEA, http://biocc.hrbmu.edu.cn/CancerSEA/goSearch).

### 2.2 Weighted gene co‐expression network analysis

The R package of “WGCNA” was used to analyze the gene co‐expression of LUAD patients receiving chemotherapy. Genes with co‐expression profiles were clustered using flasClust, and converted into an adjacency matrix and topological overlap matrix. The dynamic tree cut algorithm was used to classify genes with similar expression patterns into the same module. Module eigengenes and significance were calculated. Modules significantly related to chemotherapy response were selected as key modules.

### 2.3 Functional analysis

For the functional analysis of the key chemotherapy‐related module, pathway enrichment and biological process annotation of eigengenes within the key module were annotated by Metascape (http://metascape.org/gp/index.html) with default parameters. The top 12 significantly representative terms were selected and converted into a network layout. The LinkOmics database (http://www.linkedomics.org/) was used for the functional analysis of the ICE1 co‐expressed genes.

### 2.4 Construction of ceRNA network

The ceRNA network was established based on the genes in the key chemotherapy‐related module. The “GDCRNATools” package in R was applied to predict lncRNA–miRNA pairs and miRNA–mRNA pairs. Cytoscape software was used for ceRNA network visualization.

### 2.5 Overall survival and risk score model analysis

The overall survival analysis of SVIL‐AS1, miR‐103a‐3p, and ICE1 was performed according to TCGA and Kaplan–Meier plotter databases. Risk score based on SVIL‐AS1, miR‐103a‐3p, and ICE1 was calculated as the following method: risk score = (*β*1 * RNA1 expression level) + (*β*2 * RNA2 expression level) + (*β*3 * RNA3 expression level). Time‐dependent receiver operating characteristic (ROC) curve was generated using the “survival‐ROC” package in R. The area under the curve (AUC) was calculated to evaluate the accuracy of prognosis prediction.

### 2.6 Construction of DDP‐resistant cells

Human LUAD cell lines, A549 and H1975, were purchased from ATCC. Cells were cultured in RPMI‐1640 medium (Invitrogen, CA, USA) with 10% fetal bovine serum (FBS; Gibco, CA, USA) at 37℃ in 5% CO_2_. A549 and H1975 cells were treated with gradient concentration of DDP (Sigma‐Aldrich, MO, USA) by intermittent stepwise selection protocol to construct DDP‐resistant cells (A549/DDP and H1975/DDP). A549/DDP and H1975/DDP cells were exposed to 5 μM DDP at 37℃ in 5% CO_2_.

### 2.7 Cell transfection

SVIL‐AS1 sequence was cloned into pcDNA3.1 plasmid (Invitrogen). miR‐103a mimic, miR‐NC mimic, miR‐103a inhibitor (in‐miR‐103a), and inhibitor control (si‐miR‐NC) were purchased from GenePharma (Shanghai, China). si‐ICE1 and si‐control were purchased from GenePharma. Cell transfection was carried out with Lipofectamine 3000 (Invitrogen) according to the manufacturer's instruction.

### 2.8 Reverse transcription‐quantitative PCR

Total RNA of cells was extracted using TRIzol (Invitrogen). Reverse transcription of cDNA was performed using Prime Script TM RT reagent kit (Takara, Dalian, China). SYBR Premix Ex Taq Kit (Takara) was used for reverse transcription‐quantitative (RT‐qPCR) reaction. GAPDH and U6 were used as endogenous controls. The relative expression of genes was calculated by 2^−ΔΔCt^ method. The primer sequences were as follows: miR‐103a‐3p forward, 5′‐ACA CTCCAGCTGGGAGCAGCATTGTACAGGG‐3′ and reverse, 5′‐TGGTGTCGTGGAGTCG‐3′; U6 forward, 5′‐CTCGCTTCGGCAGCA CA‐3′ and reverse, 5′‐AACGCTTCACGAATTTGCGT‐3′; SVIL‐AS1 forward 5′‐ TAGGATGGACGAGGACGTGT‐3′ and reverse, 5′‐GACTGTCTGCCTCGTGTTCA‐3′; ICE1 forward 5′‐ATTTCGGGAAACGACTGGCT‐3′ and reverse, 5′‐ACAGAGTCAGAGCACAAGGC‐3′; and GAPDH forward, 5′‐GGCACAGTCAAGGCTGAGAATG‐3′ and reverse, 5′‐ATGGTGGTG AAGACGCCAGTA‐3′.

### 2.9 Cell counting kit‐8

For cell proliferation analysis, A549/DDP and H1975/DDP cells were seeded into 96‐well plates with 5 × 10^5^ cells per well, and cultured (with 50 μM DDP) for 0, 24, 48, and 72 h. Then, 10 μl of Cell counting kit (CCK‐8) solution (Takara) was added for another 4 h. The absorbance at 450 nm was detected by a microplate reader (Bio‐Rad, Hercules, CA, USA). For IC50 analysis, A549, H1975, A549/DDP, and H1975/DDP cells were treated with 10, 20, 40, 60, 80, and 100 μM DDP for 48 h. The dose–response curve was charted to calculate the IC50 value.

### 2.10 Dual‐luciferase reporter assay

The potential binding sites of SVIL‐AS1/miR‐103a‐3p and miR‐103a‐3p/ICE1 pairs were predicted using StarBase v2.0 (http://starbase.sysu.edu.cn/) and TargetScan (http://www.targetscan.org/). The wild type (WT) and mutant type (MT) sequences of SVIL‐AS1 and ICE1 were synthesized by GenePharma, and cloned into pGL3 plasmid (Promega Corporation, Madison, WI, USA). The recombinant reporter plasmids were co‐transfected with miR‐103a‐3p mimic or NC mimic into A549/DDP and H1975/DDP cells using Lipofectamine 3000 (Invitrogen). After 24 h incubation, the luciferase activity was measured using dual‐luciferase reporter assay system (Promega).

### 2.11 Western blot assay

A549/DDP and H1975/DDP cells were transfected with si‐control, si‐ICE1, or pcDNA3.1‐SVIL‐AS1 for 48 h. Then, the total protein of cells was extracted using RIPA buffer (Thermo Fisher Scientific, MA, USA). The protein concentration was measured using a BCA protein assay kit (Thermo Fisher Scientific). Protein (40 µg) was separated by sodium dodecyl sulfate‐polyacrylamide gel electrophoresis and transferred to a polyvinylidene difluoride membrane. After blocked with 5% nonfat milk, the membranes were incubated with primary antibodies at 4℃ overnight and then incubated with goat anti‐mouse antibody (1:1000, #56970; Cell Signaling) at room temperature for 2 h. GAPDH was used as an internal reference. ECL chemiluminescent detection system and Fluor Chem 2.0 software were used for immunoblot visualization and calculation. The primary antibodies were as follows: anti‐γ‐H2AX (1:500, 05‐636; Millipore) and anti‐GAPDH (1:1000, #5174; Cell Signaling).

### 2.12 Statistical analysis

Data were presented as mean ± standard deviation. Statistical tests were performed with R. *p* < 0.05 was considered to be statistically significant.

## 3 RESULTS

### 3.1 Identification of the key chemotherapy‐related module in LUAD

**FIGURE 1:**
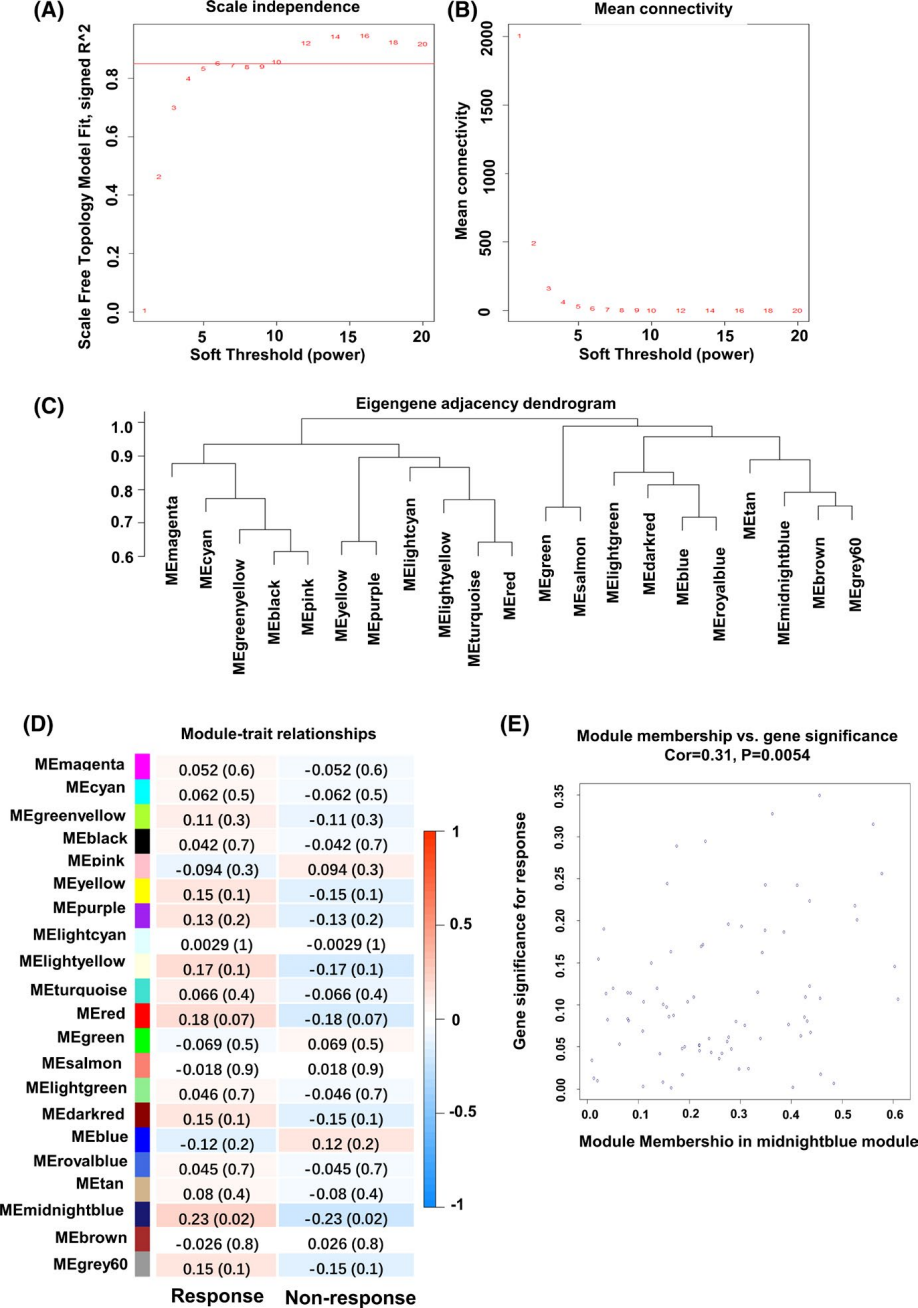
WGCNA of LUAD patients receiving chemotherapy in TCGA. (A,B) Scale‐free topology fit (A) and mean connectivity (B) for various soft threshold power. (C) Eigengene adjacency dendrogram of the merged module. (D) Heatmap of module–traits relationship. The correlation and *p* value (numbers in parentheses) of each module were shown in each table. (E) Correlation of eigengenes in midnightblue module with chemotherapy. LUAD, lung adenocarcinoma; WGCNA, weighted gene co‐expression network analysis

To explore the genes that influence LUAD chemotherapy, WGCNA was performed on the expression profile data of LUAD patients receiving chemotherapy in TCGA database. The *β* value at the scale‐free topology fitting index *R* ^2^ = 0.85 was selected as the soft threshold power to ensure that the network conforms to the scale‐free distribution. As shown in Figure 1A, *β* = 6 was selected as the soft threshold power. Mean connectivity analysis of various soft threshold power suggested that it is reasonable to choose *β* = 6 as the soft threshold power (Figure 1B). The dynamic tree pruning method was used to construct co‐expression modules. Modules with more than 30 genes were chosen for further analysis. The degree of dissimilarity among different modules was analyzed by constructing eigengene adjacency dendrogram and heatmap. Modules with dissimilarity coefficient <0.6 were merged. A total of 21 merged modules were obtained (Figure 1C).

To explored the key module related to chemotherapy, 133 LUAD patients receiving chemotherapy were classified into two types of clinical traits, “response to chemotherapy” and “non‐response to chemotherapy.” By calculating the correlation between eigengenes and the clinical traits, we found that only midnightblue module was significantly correlated with chemotherapy, while the other 20 modules were not significantly correlated with chemotherapy (Figure 1D). There were 71 genes in midnightblue module, which were significantly associated with chemosensitivity in LUAD (Cor = 0.31, *p* = 0.0054) (Figure 1E).

### 3.2 Functional analysis of genes in midnightblue module

**FIGURE 2:**
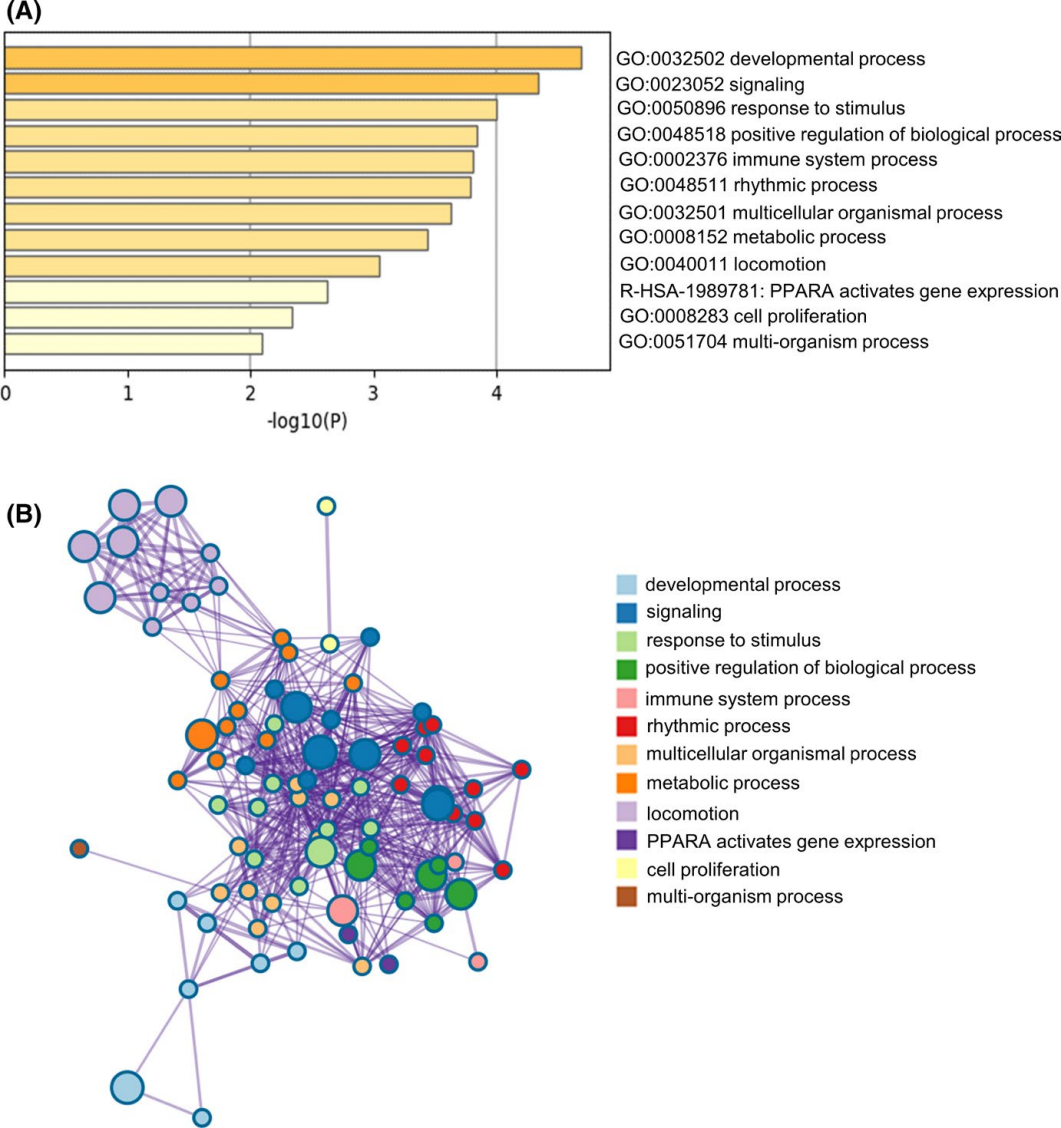
Functional analysis of genes in midnightblue module via Metascape. (A) The top 12 significantly representative terms. (B) The similarities of enriched terms for eigengenes in midnightblue module. Each term was represented by a circular node, the size of which was proportional to the number of genes in the term

The 71 genes in midnightblue module were used for functional analysis. The result revealed that these genes were closely associated with “response to stimulus,” “immune system process,” “PPARA activates gene expression,” and “cell proliferation” (Figure 2A). The similarities of enriched terms for genes in midnightblue module were shown in Figure 2B.

### 3.3 Construction of the ceRNA network of genes in midnightblue module

**FIGURE 3:**
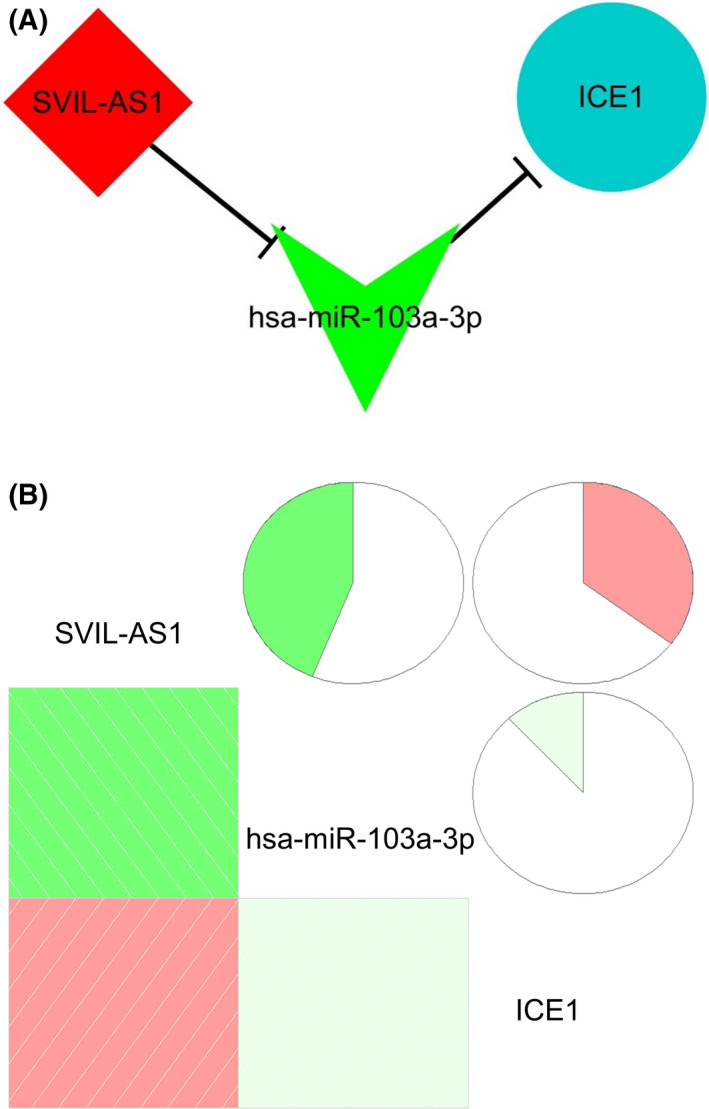
ceRNA network based on the eigengenes of midnightblue module. (A) SVIL‐AS1/miR‐103a‐3p/ICE1 axis. (B) Correlation analysis of SVIL‐AS1, miR‐103a‐3p, and ICE1 expression. ceRNA, competing endogenous RNA

To further explore the relationship of genes in the key chemotherapy‐related module, ceRNA network was established based on all of the 71 genes in midnightblue module. Interestingly, a ceRNA axis containing SVIL‐AS1/miR‐103a‐3p/ICE1 was constructed (Figure 3A). We explored the correlation of expression levels among the three genes in the ceRNA axis based on TCGA database. As shown in Figure 3B, the expression level of SVIL‐AS1 was negatively correlated with miR‐103a‐3p, but positively correlated with ICE1. There was a negative correlation between miR‐103a‐3p and ICE1 (Figure 3B).

### 3.4 Prognostic signature of SVIL‐AS1/miR‐103a‐3p/ICE1 axis

**FIGURE 4:**
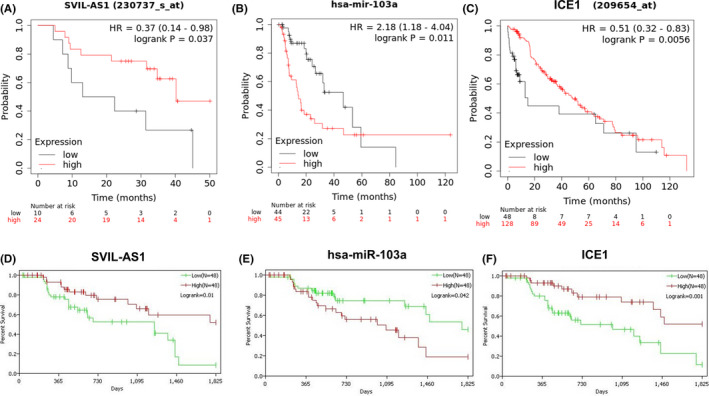
Overall survival analysis of SVIL‐AS1, miR‐103a‐3p, and ICE1 based on Kaplan–Meier plotter database (A–C) and TCGA database (D–F). TCGA, The Cancer Genome Atlas

Prognosis analysis of LUAD patients receiving chemotherapy in Kaplan–Meier plotter database showed that high expression of miR‐103a‐3p corresponded to unsatisfactory prognosis, while low expression of SVIL‐AS1 and ICE1 corresponded to unsatisfactory prognosis (Figure 4A–C). Results of TCGA database analysis were consistent with Kaplan–Meier plotter database (Figure 4D–F).

**FIGURE 5:**
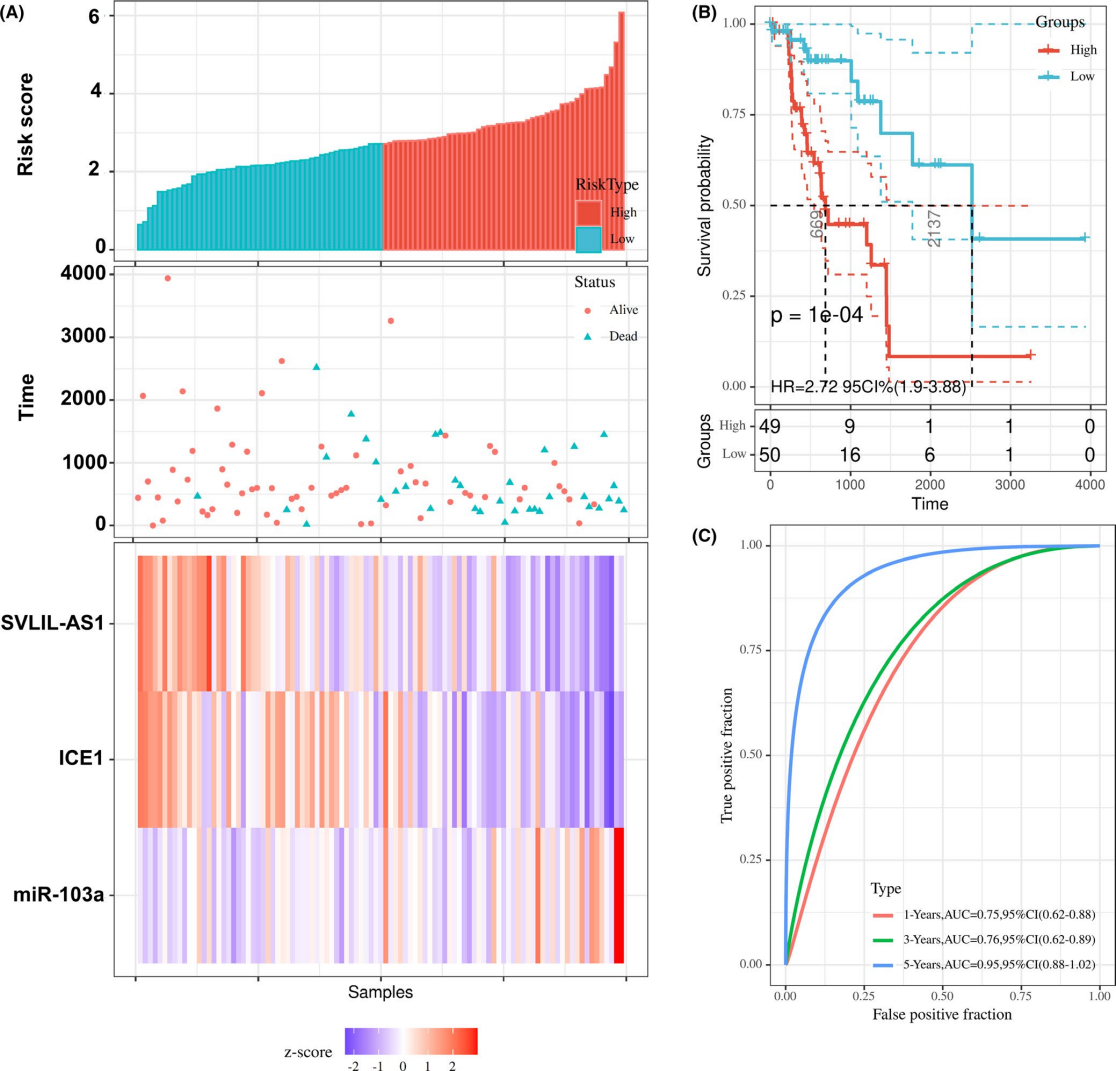
Multivariate survival analysis of SVIL‐AS1/miR‐103a‐3p/ICE1 axis. (A) Risk score, survival time, and SVIL‐AS1/miR‐103a‐3p/ICE1 expression of LUAD patients. (B) Survival probability of patients in high‐risk and low‐risk groups. (C) Time‐dependent ROC curve. LUAD, lung adenocarcinoma; ROC, receiver operating characteristic

In the multivariate survival analysis, SVIL‐AS1, miR‐103a‐3p, and ICE1 were included in the model to estimate the risk score. Patients were divided into high‐risk and low‐risk groups. The relationship between patient survival time and risk score was presented in Figure 5A. SVIL‐AS1 and ICE1 were low expressed in the high‐risk group, while miR‐103a‐3p was high expressed in the high‐risk group (Figure 5A). The survival probability of the high‐risk group was obviously lower than that in the low‐risk group (Figure 5B). ROC curves showed that the 1‐, 3‐, and 5‐year AUC was 0.75, 0.76, and 0.95, indicating satisfactory prognostic prediction efficacy (Figure 5C).

### 3.5 Validation of SVIL‐AS1/miR‐103a‐3p/ICE1 axis in LUAD cells

**FIGURE 6:**
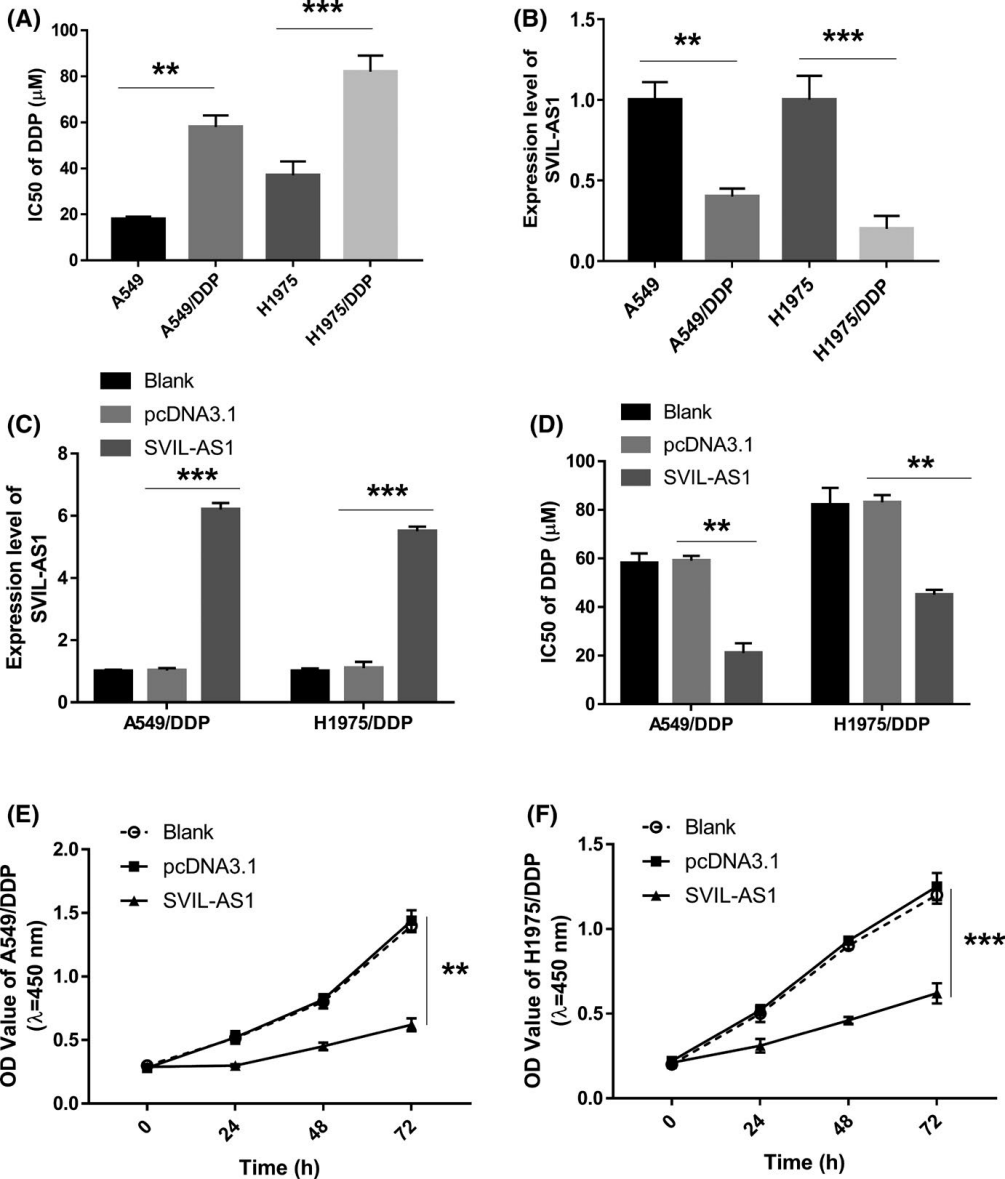
Effect of SVIL‐AS1 overexpression on DDP‐resistance and proliferation in A549/DDP and H1975/DDP cells. (A) IC50 value of DDP‐sensitive cells (A549 and H1975) and DDP‐resistant cells (A549/DDP and H1975/DDP). (B) Relative expression level of SVIL‐AS1 in A549, H1975, A549/DDP, and H1975/DDP cells. (C) Relative expression level of SVIL‐AS1 in A549/DDP and H1975/DDP cells transfected with pcDNA3.1 or pcDNA3.1‐SVIL‐AS1. (D) Effect of SVIL‐AS1 overexpression on IC50 of DDP in A549/DDP and H1975/DDP cells. (E,F) Effect of SVIL‐AS1 overexpression on cell proliferation in A549/DDP (E) and H1975/DDP (F) cells. **p < 0.01; ***p < 0.001. DDP, cisplatin

To further testify the role of SVIL‐AS1/miR‐103a‐3p/ICE1 axis in DDP resistance of LUAD cells, DDP‐resistant cells (A549/DDP and H1975/DDP cells) were constructed. As indicated in Figure 6A, the IC50 value of DDP‐resistant cells was increased compared with the DDP‐sensitive cells. The expression of SVIL‐AS1 in DDP‐resistant LUAD cells was significantly lower than that of the corresponding DDP‐sensitive cells (Figure 6B). Subsequently, SVIL‐AS1 was overexpressed in A549/DDP and H1975/DDP cells (Figure 6C). The IC50 value of DDP in A549/DDP and H1975/DDP cells was significantly reduced in the SVIL‐AS1 overexpression group (Figure 6D). Overexpression of SVIL‐AS1 significantly inhibited the cell proliferation in A549/DDP and H1975/DDP cells (Figure 6E–F).

**FIGURE 7:**
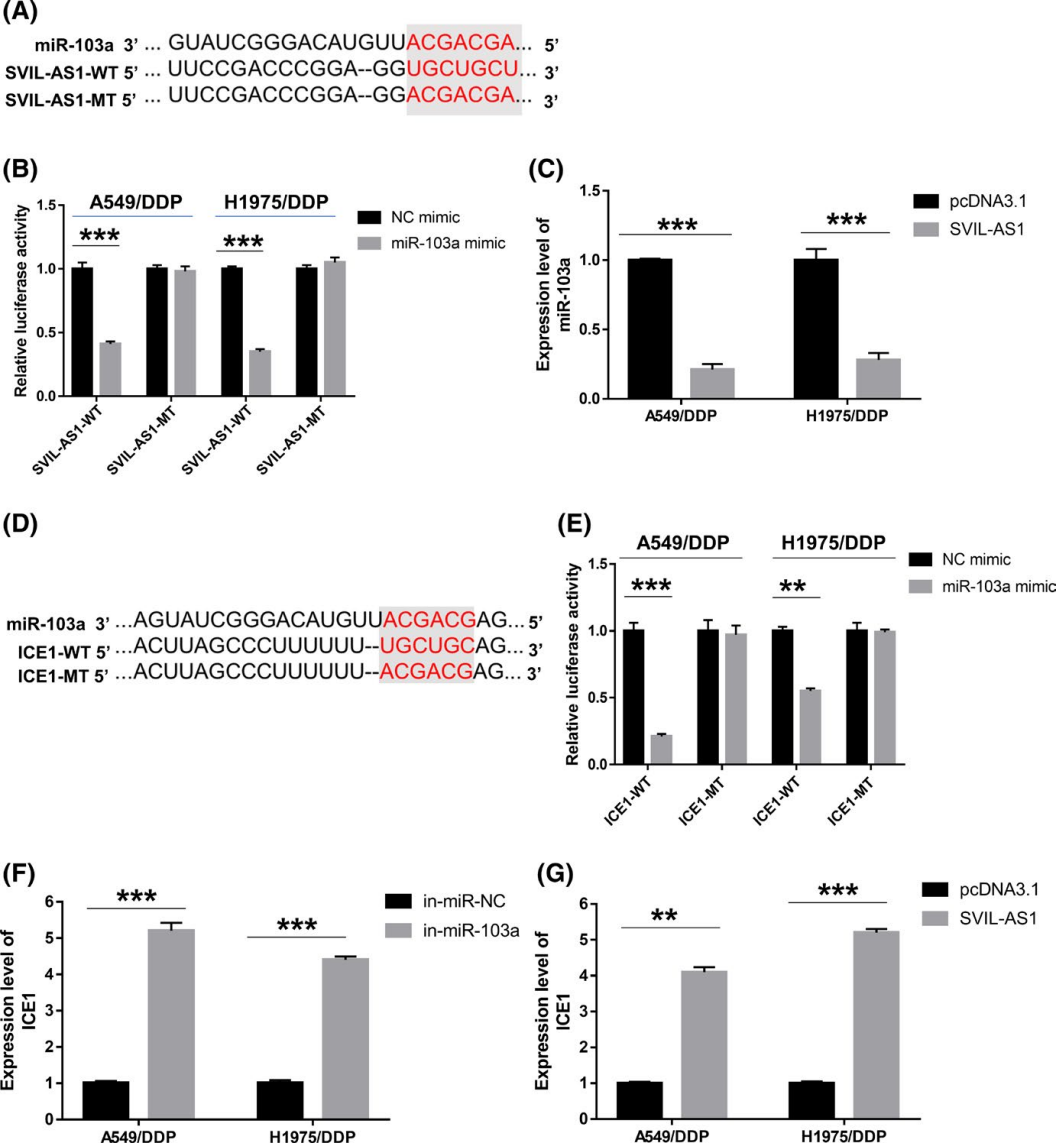
Targeting relationship of SVIL‐AS1/miR‐103a‐3p and miR‐103a‐3p/ICE1 pairs. (A) The predicted binding sites of miR‐103a at 3′‐UTR of SVIL‐AS1. (B) Luciferase activity of SVIL‐AS1‐WT or SVIL‐AS1‐MT in cells transfected with NC mimic or miR‐103a mimic. (C) Relative expression level of miR‐103a in A549/DDP and H1975/DDP cells transfected with pcDNA3.1 or pcDNA3.1‐SVIL‐AS1. (D) The predicted binding sites of miR‐103a at 3′‐UTR of ICE1. (E) Luciferase activity of ICE1‐WT or ICE1‐MT in cells transfected with NC mimic or miR‐103a mimic. (F) RT‐qPCR analysis of ICE1 expression in A549/DDP and H1975/DDP cells transfected with inhibitor control (in‐miR‐NC) or miR‐103a inhibitor (in‐miR‐103a). (G) RT‐qPCR analysis of ICE1 expression in A549/DDP and H1975/DDP cells transfected with pcDNA3.1 or pcDNA3.1‐SVIL‐AS1. **p < 0.01; ***p < 0.001. RT‐qPCR, reverse transcription‐quantitative PCR

The predicted binding sites of miR‐103a at 3′‐UTR of SVIL‐AS1 were shown in Figure 7A. miR‐103a mimic significantly downregulated the relative luciferase activity in SVIL‐AS1‐WT cells, but had no significant effect on the luciferase activity of SVIL‐AS1‐MT cells (Figure 7B). The results of RT‐qPCR showed that overexpression of SVIL‐AS1 repressed miR‐103a expression in A549/DDP and H1975/DDP cells (Figure 7C). Similarly, the target binding between miR‐103a and ICE1 was verified by dual‐luciferase reporter assay, as evidenced by the decreased luciferase activity in cells co‐transfected with ICE1‐WT and miR‐103a mimic (Figure 7D,E). Besides, ICE1 expression was facilitated by miR‐103a silencing or SVIL‐AS1 overexpression in A549/DDP and H1975/DDP cells (Figure 7F,G). These results suggested that SVIL‐AS1 promoted ICE1 expression through sponging miR‐103a.

**FIGURE 8:**
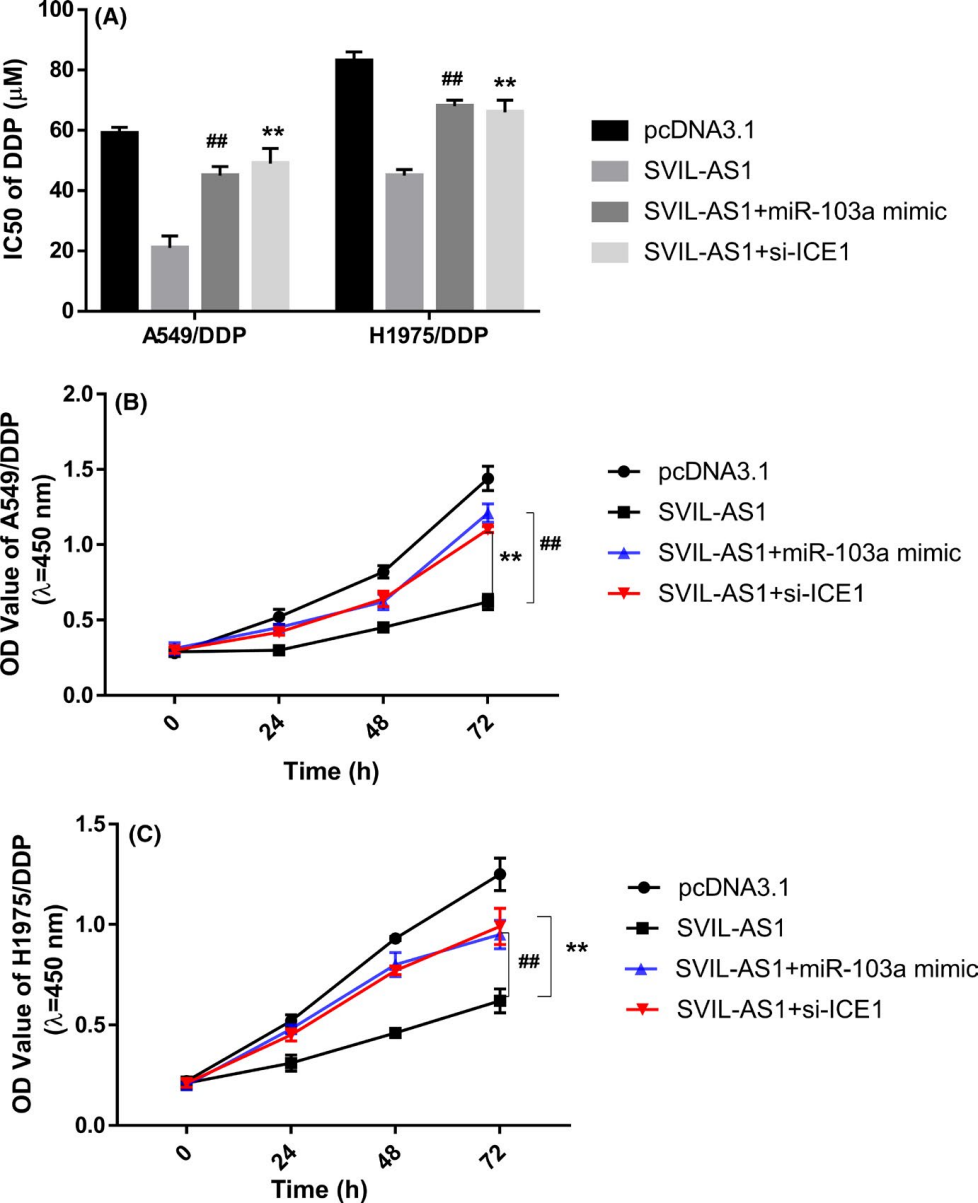
Rescue experiments of SVIL‐AS1 overexpression in A549/DDP and H1975/DDP cells. (A) IC50 of DDP in A549/DDP and H1975/DDP cells transfected with pcDNA3.1, pcDNA3.1‐SVIL‐AS1, co‐transfected with pcDNA3.1‐SVIL‐AS1 and miR‐103a mimic, or co‐transfected with pcDNA3.1‐SVIL‐AS1 and si‐ICE1. (B,C) CCK‐8 assay of cell proliferation in A549/DDP (B) and H1975/DDP (C) cells transfected with pcDNA3.1, pcDNA3.1‐SVIL‐AS1, co‐transfected with pcDNA3.1‐SVIL‐AS1 and miR‐103a mimic, or co‐transfected with pcDNA3.1‐SVIL‐AS1 and si‐ICE1. **p < 0.01, SVIL‐AS1+si‐ICE1 versus SVIL‐AS1; ^##^ *p* < 0.01, SVIL‐AS1+miR‐103a mimic versus SVIL‐AS1. DDP, cisplatin; CCK‐8, cell counting kit‐8

Additionally, rescue experiments were performed in A549/DDP and H1975/DDP cells. miR‐103a overexpression or ICE1 silencing partially restored the decrease of IC50 caused by SVIL‐AS1 overexpression (Figure 8A). Consistently, CCK‐8 assay showed that SVIL‐AS1 overexpression mediated the decrease of cell proliferation improved by miR‐103a overexpression or ICE1 silencing in A549/DDP and H1975/DDP cells (Figure 8B,C).

### 3.6 Functional analysis and validation of ICE1 co‐expressed genes

**FIGURE 9:**
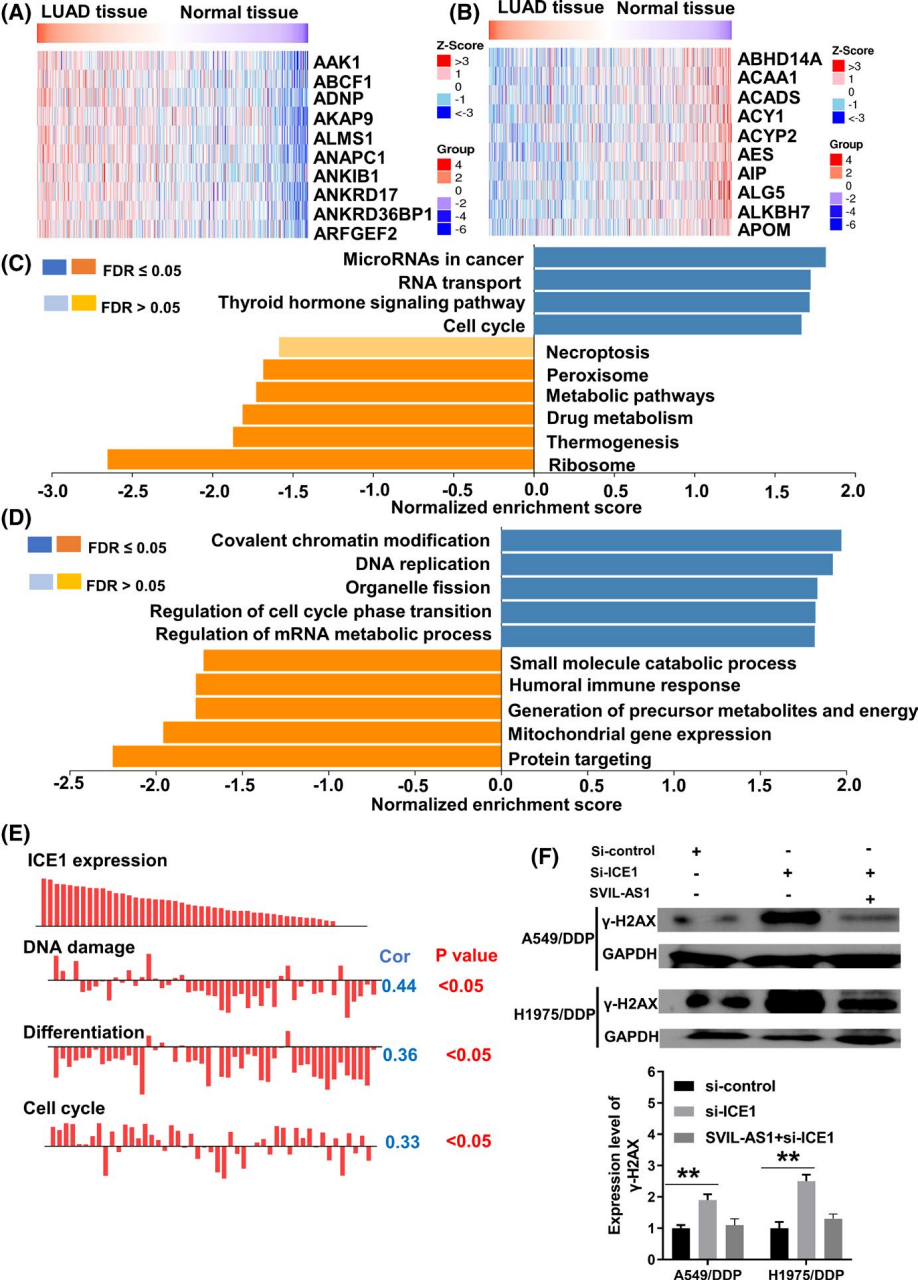
Identification and functional analysis of ICE1 co‐expressed genes in LUAD. (A,B) Heatmap of the top 10 genes positively (A) and negatively (B) correlated with ICE1 expression. (C) GO analysis of ICE1 co‐expressed genes in LUAD. Blue, GO terms for genes positively correlated with ICE1 expression. Orange, GO terms for genes negatively correlated with ICE1 expression. (D) KEGG analysis of ICE1 co‐expressed genes in LUAD. Blue, GO terms for genes positively correlated with ICE1 expression. Orange, GO terms for genes negatively correlated with ICE1 expression. (E) Single‐cell sequencing analysis was performed using CancerSEA database. (F) Western blot was applied to detect the level of γ‐H2AX in A549/DDP and H1975/DDP cells transfected with si‐control, si‐ICE1, or pcDNA3.1‐SVIL‐AS1. **p < 0.01. GO, Gene Ontology; KYOTO, Kyoto Encyclopedia of Genes and Genomes; LUAD, lung adenocarcinoma

To further investigate how the SVIL‐AS1/miR‐103a/ICE1 axis regulates chemoresistance in LUAD, the co‐expressed genes of ICE1 in LUAD were screened using the LinkOmics database. The results showed that a total of 11,200 genes were significantly correlated with ICE1 expression, including 5915 positively correlated genes and 5285 negatively correlated genes. The top 10 positively and negatively correlated genes were indicated in Figure 9A,B, respectively. Gene Ontology (GO) annotation indicated that genes positively associated with ICE1 expression were related to cell cycle, while genes negatively associated with ICE1 expression were related to drug metabolism (Figure 9C). Kyoto Encyclopedia of Genes and Genomes results were consistent with GO annotation, indicating that ICE1 co‐expressed genes were positively correlated with cell cycle‐related pathways, such as “regulation of cell cycle phase transition” and “organelle fission,” but negatively correlated with drug metabolism‐related pathways such as “small molecule catabolic process” (Figure 9D). Furthermore, the function of ICE1 was further analyzed based on the CancerSEA database. As shown in Figure 9E, ICE1 expression was positively correlated with DNA damage (Cor = 0.44, *p* < 0.05), differentiation (Cor = 0.36, *p* < 0.05), and cell cycle (Cor = 0.33, *p* < 0.05). To verify the above results, the protein expression level of DNA damage marker γ‐H2AX was measured. Knockdown of ICE1 upregulated γ‐H2AX expression, while overexpression of SVIL‐AS1 restored the effects of ICE1 knockdown (Figure 9F).

## 4 DISCUSSION

Chemotherapy is beneficial for survival in LUAD patients. However, chemoresistance usually impedes the effectiveness of treatment and causes tumor recurrence. At present, the therapeutic effect of LUAD is still unsatisfactory. The 5‐year survival rate of LUAD is only about 15%.^4^ The causes of chemoresistance are complex. The mechanism of LUAD chemoresistance is not fully understood. In the present report, SVIL‐AS1/miR‐103a/ICE1 axis was found to play an important role in LUAD chemoresistance by combining WGCNA with ceRNA network construction.

Weighted gene co‐expression network analysis is a bioinformatics algorithm that analyzes the correlations of gene expression in large‐scale datasets and correlates gene modules with clinical traits.^9^, ^10^ WGCNA is widely used in the research of cancer occurrence and development. In this study, WGCNA analysis was performed on the data of LUAD patients receiving chemotherapy from TCGA database. A total of 21 modules were obtained, among which only midnightblue module showed significant correlation with chemosensitivity. Functional annotations indicated that genes in midnightblue module were significantly enriched in “response to stimulus,” “immune system process,” “PPARA activates gene expression,” and “cell proliferation.” These metabolic processes are closely related to cancer development and chemoresistance. Immune system can recognize and eliminate tumor cells in the early stage of cancer.^23^, ^24^, ^25^, ^26^ However, when tumor cells escape from immune surveillance and form clusters through cell proliferation, the ability of immune system to monitor and eliminate tumor cells is diminished, leading to mutual adaptation of tumor cells and the immune system.^23^, ^24^, ^25^, ^26^ Chemotherapeutic drugs act as an external stimulus to induce immune system to exert its antitumor immune response.^27^ Peroxisome proliferator‐activated receptor alpha (PPARA) is involved in the regulation of cancer progression. For example, lncRNA LINC00467 regulates the progression of hepatocellular carcinoma by regulating the expression of miR‐9‐5p/PPARA.^28^ Therefore, we speculated that genes in midnightblue module may play beneficial roles in the survival of LUAD patients receiving chemotherapy.

Competing endogenous RNA is an important mechanism for lncRNA to regulate cancer progression and chemoresistance.^29^, ^30^ To better understand the regulation mechanism of genes on chemoresistance, a ceRNA axis (SVIL‐AS1/miR‐103a/ICE1) was constructed based on the key chemotherapy‐related module. Kaplan–Meier survival analysis of genes in SVIL‐AS1/miR‐103a/ICE1 axis showed that in the LUAD patients receiving chemotherapy, high expression of SVIL‐AS1 and ICE1 corresponded to a better prognosis, while high expression of miR‐103a corresponded to a poor prognosis. There are limited studies on the role and mechanisms of SVIL‐AS1 in cancer. SVIL‐AS1 is abnormally expressed in NSCLC. The promoter of SVIL‐AS1 is usually hypermethylated in NSCLC.^31^ Results of this study indicated that SVIL‐AS1 was downregulated in DDP‐resistant LUAD cells. Overexpression of SVIL‐AS1 reduced proliferation and IC50 of DDP in LUAD cells. SVIL‐AS1 inhibited LUAD chemoresistance by acting as a sponge for miR‐103a and upregulating ICE1 expression. miR‐103a plays an important role in lung cancer progression and chemoresistance.^32^, ^33^, ^34^, ^35^ miR‐103 acts as an oncogene in NSCLC by targeting lncRNA TRHD‐AS1, KLF4, and KLF7.^32^, ^36^ In DDP‐resistant NSCLC cells, the expression of miR‐103a‐3p is upregulated.^37^ miR‐103a‐3p activates ERK signaling pathway and leads to DDP resistance in NSCLC.^37^ In addition, miR‐103a‐3p accelerates DDP resistance in NSCLC cells via Bak1 downregulation.^38^ In this study, the direct targeting relationship of SVIL‐AS1/miR‐103a and miR‐103a/ICE1 was verified. miR‐103a overexpression and ICE1 knockdown abolished the inhibitory effect of SVIL‐AS1 on IC50 and cell proliferation.

ICE1 is an interactor of little elongation complex ELL subunit 1. Hiroki Sasaki et al. have shown that ICE1 is abnormally expressed in gastric cancer.^39^ However, the function and regulation mechanism of ICE1 in LUAD has not been reported. To investigate the molecular mechanisms by which SVIL‐AS1/miR‐103a/ICE1 axis regulates chemoresistance in LUAD, we analyzed the signaling pathways and functions of ICE1 co‐expressed genes in LUAD using the LinkOmics database, and performed a more in‐depth analysis through the CancerSEA database. The results showed that ICE1 was significantly positively correlated with cell cycle transition, but negatively correlated with the metabolism of small molecule drugs in LUAD. Further single‐cell sequencing analysis showed that ICE1 showed a positive correlation with DNA damage. Therefore, we hypothesized that SVIL‐AS1/miR‐103a‐3p/ICE1 axis may inhibit chemotherapeutic drug metabolism and promote DNA damage, thereby enhancing chemosensitivity. Further experimental verification also shown that SVIL‐AS1/miR‐103a‐3p/ICE1 axis can enhance DNA damage caused by chemotherapeutic agents. However, the molecular mechanisms of ICE1 in regulating DNA damage of lung cancer cells still need to be further studied.

## 5 CONCLUSION

In summary, the present study identified a novel ceRNA axis (SVIL‐AS1/miR‐103a/ICE1) that regulated LUAD chemoresistance. SVIL‐AS1, miR‐103a, and ICE1 can be used as independent risk factors for the prognosis of LUAD patients with chemotherapy. SVIL‐AS1/miR‐103a/ICE1 axis constructed in this research is helpful to advance the study on the mechanism of chemoresistance in LUAD. This study improved the molecular mechanism of LUAD chemoresistance, and provided a theoretical basis for improving LUAD chemotherapy.

## ETHICAL APPROVAL STATEMENT

Our study is based on open‐source database, the patients involved in the database have obtained ethical approval. There are no ethical issues and other conflict of interest.

## CONFLICT OF INTEREST

The authors declare that they have no competing interest.

## Data Availability

The original contributions presented in the study are included in the article and further enquiries can be directed to the corresponding author (Email: tangjf1969@163.com).
